# Pediatric Robotic Surgery in South America: Advantages and Difficulties in Program Implementation

**DOI:** 10.3389/fped.2019.00094

**Published:** 2019-03-28

**Authors:** Juan M. Moldes, Francisco Ignacio de Badiola, Roberto Luis Vagni, Pedro Mercado, Virginia Tuchbaum, Marcos G. Machado, Pedro José López

**Affiliations:** ^1^Department Pediatric Surgery, Hospital Italiano de Buenos Aires, Buenos Aires, Argentina; ^2^Department of Urology, Faculdade de Medicina, Universidade de São Paulo, Hospital das Clínicas, São Paulo, Brazil; ^3^Department Pediatric Urology, Hospital Exequiel Gonzalez Cortés y Clínica Alemana, Santiago, Chile

**Keywords:** robotic surgery, pediatric, minimally invasive surgery, pediatric urology, South America

## Abstract

Robotic assisted laparoscopic surgery is gaining popularity around the world due to its vast benefits. Although it has been established mainly in developed countries, in South America the robotic programs have become more popular, but its growth is clearly slower. Information about robotic pediatric surgery program in Brazil, Chile, Uruguay, and Argentina was collected through e-mail surveys. Results were analyzed and compared to worldwide information about robotic surgery. Due to the wide social, economical, and technological gap between hospitals in South America, it is hard to develop a proper pediatric robotic surgery program. The main obstacles in those four countries appear to be a combination of high purchase costs and equipment maintenance, lack of financial coverage of the procedure by insurance companies and the absence of significant benefits proved in pediatrics in relation to laparoscopic surgery. The pediatric specialties are in the process of making and implementing robotic programs supported by the evident development in adult specialties. However, pediatric robotic surgery in Brazil, Chile, Uruguay and Argentina do not seems to share that growth.

## Introduction

Robotic assisted surgery is one of the most advanced forms of Minimally Invasive Surgery. It has been used worldwide on a broad range of medical specialties since the 1990s, evolving rapidly evolved since then (1). In the pediatric surgery field, it has been mainly adopted on urologic procedures, where more complex surgeries which require extreme precision are performed. However, there are few reports of pediatric urology procedures done with this technology compared to what is published related to adult's surgery.

Pediatric robotic surgery has undergone significant growth since its first application in 2002 (2, 3). The first pediatric robotic procedure performed at most centers was the robotic assisted laparoscopic pyeloplasty. The relatively high incidence of ureteropelvic junction obstruction combined with surgeon familiarity with laparoscopic pyeloplasty made it a natural first robotic procedure. Since then, it has become more commonly performed accounting for 11–12.6% of pyeloplasties performed in the USA by 2009 (4), and it accounts for about 40% of cases nowadays (5–7).

Ureteral reimplantation, both intra or extravesical approach, robotic assisted ureteroureterostomy for the treatment of ectopic ureter and ureterocele have become more frequently performed according to recent publications. On the other hand there are an increasing number of reports of complex reconstructive procedures, such as urinary incontinence treatment with procedures on the bladder neck, bladder augmentation, and continent urinary stoma (5, 8, 9).

While extirpative procedures were described, they are not becoming popular with a robotic approach, likely due to that they are relatively easy to master with a pure laparoscopic approach, making it less cost—effective (2). The progress of robotic assisted surgery is predominant in developed countries, however, in Latin America, regardless of its limitations, it is growing.

Our objective is to describe the current situation of robotic pediatric surgery in four countries of South America, describing the limitations and difficulties that have been faced on the implementation of a long term pediatric robotic program.

## Methods

Information about robotic pediatric surgery program were collected in Brazil, Chile, Uruguay, and Argentina. The following data was collected: number of active programs in each country, year the program started working, number of surgeons and pediatric surgeons trained in robotics, the estimated number of surgeries performed during the program and costs of the surgery and entity responsible for the payment.

Data through a survey regarding pitfalls of the pediatric robotic surgery program was also requested among centers and surgeons performing pediatric robotic surgery.

## Results

In the region where the survey was conducted, we found a total of 52 active robotic equipment. Brazil, with 40 robots, is the country that has experienced the major progress in the area. In relation to its general population and the number of robots, it appears that Chile is the country with the best coverage of its population with 1 robot every 2.1 million people, followed by Uruguay with 1 every 3.4, Brazil with 1 every 5.1 million people and Argentina with 1 robot every 14.6 million people (Table 1). In relation to the pediatric population younger than 14 years old, the proportion is similar to the adults (Table 2).

**Table 1:** Number of Robots related to the country population.

**Country/region**	**Robots**	**Population (million)**	**Robot/people (million)**
Brazil	40	207	1/5.1
Argentina	3	44	1/14.6
Chile	8	17	1/2.1
Uruguay	1	3	1/3.1
USA	2,862	323	1/0.11
Europe	742	741	1/0.99
Asia	579	4,463	1/7.7

**Table 2:** Population under 14 years old in year 2017.

	**Population 2017**	**% kids under 14**	**Total**
Brazil	207,660,929	22.79	46,899,407
Argentina	44,098,971	24.7	10,892,445
Chile	17,373,831	20.27	3,577,092
Uruguay	3,456,750	20.44	684,982

The location of the robots is similar in the four countries where the systems are mostly gathered in one or two big cities. In Chile and Uruguay, 100% of the equipment's are in the capital cities (Santiago de Chile and Montevideo, respectively), while in Argentina the 66% are in Buenos Aires. In Brazil, 75% are located in the two major cities which are Sao Paulo and Rio de Janeiro.

Regarding the surgeons and pediatric urologists accredited in robotic surgery by Intuitive® company, it has been difficult to obtain the accurate information related to Brazil because the enormous geographic area that covers that country. A total around 400 surgeons are accredited by the company, most of them trained overseas. A local training center is planned to start to work in 2019 in Rio de Janeiro. Among them, we were only able to collect information from four pediatric surgeons active in robotic surgery.

In the rest of the three countries, it is clear that Chile has the largest number of pediatric surgeons trained with 2.5 doctors per robot, followed by Argentina with 0.3 and Uruguay without any accredited one. The relationship between accredited adult and pediatric surgeons, in Chile the proportion is from 106 to 19, in Argentina from 24 to 1 and in Uruguay from 3 to 0.

It is interesting to note that the vast majority of accreditations were made in the first years after the acquisition of robotic systems (Table 3).

**Table 3:** Description of Robot programs in South America.

	**Active robots**	**Program started**	**Trained adult surgeons**	**Trained pediatric surgeons**	**Trained pediatric urologist**
Brazil	40	2008	400	2	2
Chile	8	2010	106	13	6
Argentina	3	2008	24	1	1
Uruguay	1	2011	3	0	0

In none of the four countries described are there any active training programs for pediatric surgeons and there are only two pediatric proctors accredited by Intuitive, one in Brazil and one in Chile.

The number of procedures varies a lot in the region, highlighting Brazil with more than 21,000 surgeries since the acquisition of the first robot in 2008. The average of surgeries performed by robot is between 525 and 625 procedures per system in Brazil, Chile, and Argentina. Within these described procedures the number of pediatric surgeries does not exceed 4% of the total in any country. We can also notice that the curve of use of the robot is upward in adult patients while in pediatric patients it seems to grow very slowly or even decrease (Table 4).

**Table 4:** Robot utilization by country.

	**Approx. total procedures**	**Pediatric program started**	**Approx. pediatric Q procedures**	**Approx. pediatric urologic procedures**
Brazil	21,700	2008	5	30
Chile	5,000	2010	146	50
Argentina	1,700	2008	6	12
Uruguay	50	2011	0	0

In the region, most robots are located in private health institutions. In Brazil, 6 of the 40 robotic systems and in Argentina 1 of 3 are in public health institutions while in Chile and Uruguay there are none.

In the public hospitals of South America procedures are mainly paid through foundations or government coverage. With some exceptions in the private Health Organizations, the private insurances do not cover the costs, thus the usual way to achieve coverage for this surgery is to assimilate the cost to a laparoscopic procedure and the difference is pay by the patient out of his pocket.

The type of surgery performed in pediatrics was mainly urological where the pyeloplasty represented around 60% of all pediatric urology procedures in these four countries. Complex procedures such as Renal Oncological Resection, Radical Prostatectomy for rhabdomyosarcoma, Ureterocalicostomy, Vesicoureteral Reimplantation, Nephrectomy, Heminephrectomy, and excision of prostatic utricle have also been performed.

## Discussion

Despite the huge demographic and economic contrast of South America compared to North America and Europe, robotic surgery is slowly but constantly evolving and is no longer a fantasy. From the data collected in this survey it is clear that this growth is due to adult patients, and that the use in pediatric patients is very limited with not sings of increasing in the near future.

It is interesting to see the relationship of the number of robots with the population especially if we compare it with the United States and Europe where the differences are very marked. In the Region (four countries), the proportion is 1 for every 5.2 million people and in the United States it is 1 for every 112,000 people and in Europe 1 for every 998,000 people approximately. The comparison with Asia gives a similar result with our region with 1 robot every 7.7 million people. It is possible that these differences and similarities have to do with the economic realities experienced by each region.

Robotic surgery in pediatric patients is still not cost effective everywhere. Probably, the initial doubts about acquiring the robotic technology in all the countries were mainly related to the economic factor, the high cost of the acquisition of the equipment and, especially the high cost of its maintenance. This fact takes special relevance at the time of purchasing a robot system with private fund, a situation that represents the most frequent reality in South America.

Büter et al. suggests that, before initiating a robotic program, it is necessary to know that sufficient number of cases will be performed to cover and justify the equipment costs, a situation which has been difficult to assess in our countries (10). With some exceptions, in these four countries the health insurance companies do not cover such complex technologies, so it was difficult to assess the cost of the system before buying it.

As an example, the first robotic equipment that was acquired by the Hospital Italiano de Buenos Aires, had to be purchased with a bank loan that was initially paid with the medical doctor's own funds due to the lack of support from the institution. Once the robot started to work and demonstrated the system's financial self-sustainability, the hospital took full responsibility of the loan and costs related to the robot.

The usual way to achieve coverage for the insurance company is to take care of the costs as if the surgery was performed by laparoscopy and leave the patient to pay the robotic extra fee, that in the region is between USD $ 4,000 and 6,000 per procedure. Insurance companies justify this action arguing that there is no high-level scientific evidence that shows better results than laparoscopy (11).

Around 120 procedures per year are necessary to financially support the program in our countries, however there is still controversy on the number of cases per year needed to make the robotic platform cost-effective (12).

According to the number of surgeries surveyed, it is not possible for us to support the robotic system only with pediatric patients. Following the same line, Büter et al. indicates that, due to the type of cases and volume of patients who would benefit from the use of the robot in pediatric urology, it is more realistic to be a part of a multispecialty adult robotic program in order to share costs and maximize the use of robotic console (10, 12). Therefore, it will be very difficult that a pure pediatric hospital gets a robot in the future.

The public institutions that have acquired the system have faced a dilemma in the investment of economic resources in this high-cost technology with limited application for specific pathologies and without high-level results published in the literature vs. the use of those public funds in higher basic priorities with greater impact in the treated population. The surgeries are mainly paid through foundations or government coverage and many of them had their programs temporarily or definitively interrupted mainly due to high costs. As an example, in Argentina, the Federico Abete Hospital in the province of Buenos Aires, started with two robotic systems in 2009 had to definitively interrupt the program 3 years later due to the lack of economic resources awarded to sustain the program (13).

On the other hand it is interesting to note that in those public hospitals, pediatric surgery has not had any development so we can infer that it is not only an economic problem but there are a series of others factors perhaps related to the scarcity of trained pediatric surgeons, the little support of general institutions for pediatric development or to the fact that no pediatric hospital has an exclusive robotic program or to the absence of proven advantages in pediatric patients.

The number of certain surgical procedures needed to become an expert is not well established. There are no studies that have addressed the learning curve of robotic operations for surgeons-in-training (14). Prithvi et al. estimates that 100 performed surgeries are required to obtain consistent results in pediatric urology cases and one surgery per week is needed to maintain the surgical skills and to make progress in the development of new skills (15).

With this very low number of procedures performed in Latin American Pediatric Surgery Services, almost no one has managed to surpass the number of the 20 surgeries suggested in a given time to acquire the necessary skills to take full advantage of the robot's capabilities (16, 17). May be this is another reasons why many pediatric surgeons still feel more comfortable with laparoscopic surgery, where practically all of them have loosely completed the learning curve.

This low number of robotic surgeries also makes it nearly impossible to achieve the requirement to perform and/or to become a robotic surgeon according to the standards of accreditation suggested by the Society of Urologic Robotic Surgery or other consensus, such as the SAGES-MIRA Robotic Surgery Consensus Group (18, 19).

This is clearly displayed in the statistics of 52 programs available in Argentina, Brazil, Chile, and Uruguay where there are only 16 accredited pediatric surgeons and 9 pediatric urologists.

Based on what Orvieto published in 2012, that in the reduced field of pediatric surgery, simulation appears as a crucial tool for the development of robotic skills and shorten the learning curve (16, 20). Perhaps the way to accredit in the region in pediatric surgery should be based mainly on the acquisition of laparoscopic skills combined with a more complete and defined robotic simulation program. Regarding the necessary tutorship by proctors, we could consider the proctors to be from the same homonymous adult specialties as the pediatric specialties in the same hospital providing the tutoring in order to make the final stage more accessible and lowering costs, especially considering that between 4 and 10 proctoring procedures are suggested to complete the training (15).

Due to what was previously mentioned, it is and has been difficult to set training and simulation programs for pediatric surgeons, which is evident in the fact that there are no programs in execution in any countries. Only recently is there one proctor in Chile and one in Brazil to form resources in pediatrics.

Regarding the size of the instruments, the robotic surgery is not the most minimal invasive procedure we can perform on a child today. The latest development of the laparoscopic instruments give us 3 mm instruments that are delicate and precise enough to comfortably perform most of the surgeries.

In addition to the fact that we are part of an adult surgery program and that the robotic 5-mm instruments are not as good as the 8-mm instruments, due to the space that the robotic wrist needs, is why we may have to use those for pediatric surgeries resulting on an large caliber instruments especially for young children. On top of this, when we use the robot we may need to use a fourth auxiliary port, while in laparoscopic procedures we exceptionally need an extra fourth port. This instrumental issue also results in an obstacle at the time of suggesting a minimally invasive approach (16).

On the other hand a surgeon who performs a laparoscopic Pyeloplasty with 3-mm instruments in 1 h on a 1-year-old child will probably take longer with the robot, so we can consider the advantage of using it in expert hands.

It is easier to see the robotic advantages in complex procedures, especially in those where there is a lot of reconstruction suture or dissections in very complex and/or small spaces. Even with a low number of surgeries, the complex surgery is more accessible specially for senior surgeons, who know the technical issues of the surgery, but know very little of laparoscopy. So, if we sum up the previously developed items, the indication of this technology in pediatrics may be reserved for demanding surgeries in terms of the location or complexity of the reconstruction, such as pelvic floor. On the other hand, an advantage of the robotic system technology that has made available the minimal invasive surgery to all those senior pediatric surgeons who were not interested at some stage of their career to walk the “painful” early stages of the so developing laparoscopic surgery.

If we start from the evident concept that robotic instruments are superior to any other endoscopic instruments because of its advantages in the mobility and the 3D vision, maybe we should stop trying to demonstrate that the results are at least similar than laparoscopic or open approaches and assume the fact that for very complex patients the use of the latest technology makes the surgical act easier.

## Conclusions

The high cost, the difficulty of obtain enough number of procedures to get a proper expertise and the difficulty in getting a proper robotic training in pediatrics, combined with the absence of high-level scientific evidence published in pediatric patients that demonstrates clear advantages in terms of results and complications over laparoscopy and open surgery, may be the reasons why robotic surgery cannot take off in this region. Hopefully with the new developments and broad implementation of the robotic technology it will reduce costs and increase the number of pediatric patients treated with robotic surgery.

## Data Availability

All datasets generated for this study are included in the manuscript and/or the supplementary files.

## Author Contributions

JM and PJL conceived of the presented idea. JM developed the theory and performed the computations. RV and FdB verified the analytical methods. PM encouraged VT to investigate a specific aspect and supervised the findings of this work. All authors discussed the results and contributed to the final manuscript. JM wrote the manuscript with support from MM, FdB, and RV. PJL and MM helped supervise the project. Both PM and VT contributed to the final version of the manuscript. All authors provided critical feedback and helped shape the research, analysis and manuscript.

### Conflict of Interest Statement

The authors declare that the research was conducted in the absence of any commercial or financial relationships that could be construed as a potential conflict of interest.
